# The postoperative patient-reported quality of recovery in colorectal cancer patients under enhanced recovery after surgery using QoR-40

**DOI:** 10.1186/s12885-015-1799-3

**Published:** 2015-10-26

**Authors:** Dai Shida, Kotaro Wakamatsu, Yuu Tanaka, Atsushi Yoshimura, Masahiko Kawaguchi, Sachio Miyamoto, Kyoko Tagawa

**Affiliations:** Colorectal Surgery Division, National Cancer Center Hospital, 5-1-1 Tsukiji, Cyuo-ku, Tokyo, 1040045 Japan; Department of Surgery, Tokyo Metropolitan Bokutoh Hospital, 4-23-15 Koto-bashi, Sumida-ku, Tokyo, 1308575 Japan; Department of Anesthesiology, Nara Medical University, 840 Shijo-cho, Kashihara, Nara, 6348522 Japan; Department of Anesthesiology, Tokyo Metropolitan Bokutoh Hospital, 4-23-15 Koto-bashi, Sumida-ku, Tokyo, 1308575 Japan

**Keywords:** ERAS, QoR-40, Colorectal surgery, Patient-reported outcome, Fast-track surgery

## Abstract

**Background:**

Enhanced recovery after surgery (ERAS) protocols may reduce postoperative complications and the length of hospital stay. Studies of the effectiveness of ERAS should include not only doctor-reported outcomes, but also patient-reported outcomes, in order to better estimate their impact on recovery. However, patient-reported outcomes are not commonly reported. Thus, it needs to be assessed whether early discharge from the hospital is compatible with a better outcome from the viewpoint of the patients themselves.

**Methods:**

The 40-item quality of recovery score (QoR-40) is a recovery-specific, and patient-rated questionnaire, which provides a good measurement of early postoperative recovery. Ninety-four colorectal cancer patients undergoing surgery under ERAS protocol management were asked to answer QoR-40 questionnaires preoperatively and on post-operative day (POD) 1, 3, 6 and one month after surgery.

**Results:**

The median (25^th^, 75^th^ percentiles) preoperative global QoR-40 scores as an indicator of the baseline health status, was 189 (176.75, 197). On POD1 and POD3, the scores had decreased significantly to 154 (132.5, 164.25) and 177 (161.75, 190), respectively. On POD 6, the score dramatically recovered up to 183.5 (167.9, 191), which was not significantly different from the baseline level (*p* = 0.06). The scores at 1 month after surgery were 190 (176, 197). Younger patients, compared to older patients, and rectal cancer patients, compared to colon cancer patients, had significantly lower scores on POD1.

**Conclusion:**

This study clearly demonstrated that the quality of recovery based on patient-reported outcomes is in agreement with discharge around POD6 for colorectal cancer patients under ERAS.

## Background

Enhanced recovery after surgery (ERAS) is a combination of various perioperative patient care methods that integrate evidence-based interventions which reduce surgical stress, maintain the postoperative physiological function and accelerate recovery in patients undergoing major surgery [1]. ERAS protocols have been used and adopted by a wide range of surgical specialties, and have been most extensively studied in colorectal surgery [2].

ERAS protocols are aimed primarily at improving patient early recovery, which results in shorter hospital stays without adversely affecting morbidity [3]. As a result, when evaluating ERAS programs, many studies have focused on changes in the length of hospital stay (LOS) [4, 5]. In fact, meta-analyses of randomized trials in colorectal surgery have reported a decreased LOS with ERAS compared with traditional care [6]. However, the LOS is problematic as a surrogate measure of recovery, as it is influenced by many non-clinical factors, including the patient expectations, traditions, availability of community or family support, insurance status and discharge destination [7]. In addition, the LOS is largely dependent on the discharge criteria, such as the tolerance of food, pain control, defecation and independence in basic activities of daily living, reflecting the biological function, and not on the patient’s return to baseline function. In contrast, from the patient’s perspective, recovery is defined as the absence of symptoms and the ability to perform regular activities or return to work. Thus, challenges in evaluating ERAS protocols lie in determining how best to measure its effects, besides the LOS and doctor-reported outcomes.

The quality of recovery score (QoR-40) is a recovery-specific and patient-rated questionnaire that contains 40 items measuring five dimensions: the physical comfort (12 items), emotional state (nine items), physical independence (five items), psychological support (seven items) and pain (seven items) [8]. The relationship between the quality of recovery and post-operative quality of life determined using the QoR-40 and the Short Form 36 Health Survey (SF-36), which is the *de facto* standard of measurement of quality of life was investigated in a previous study, which revealed that the QoR-40 provided a better measurement of the early postoperative recovery [9]. The QoR-40 has now been validated in various countries [10, 11].

The aim of this study of colorectal cancer patients under ERAS　was to measure how the quality of recovery changes from the standpoint of the patients themselves, in order to investigate whether early discharge is good from the standpoint of the patients’ QOL.

## Methods

### Study population

This study was approved by the Tokyo Bokutoh Metropolitan Hospital institutional review boards (IRB) and written informed consent was obtained from all patients. Ninety-four patients undergoing primary colorectal cancer surgery at Tokyo Bokutoh Metropolitan Hospital were enrolled from November 2011 to December 2012. Patients were consecutively recruited to the trial during this period. The exclusion criteria included poor Japanese comprehension or psychiatric/central nervous system disturbances precluding completion of the Japanese version of the QoR-40 [12, 13]. This study was approved by the Tokyo Bokutoh Metropolitan Hospital IRB (IRB code: 25 –Heisei23).

All patients were treated using the ERAS protocols of Tokyo Metropolitan Bokutoh Hospital [14]. The main differences between the ERAS protocols of Tokyo Metropolitan Bokutoh Hospital and traditional care are intensive pre-admission counselling, no fasting and oral nutrition during the pre- and post-operative periods, no nasogastric tube after the operation, intense use of thoracic epidural anesthesia/analgesia, avoidance of sodium/fluid overload, short incisions, intraoperative warm air body heating, a routine mobilization care pathway, stimulation of gut motility (use of oral magnesium oxide), early removal of catheters and a multidisciplinary team approach.

The patient demographic and perioperative data were also collected. These included the type of surgery, American Society of Anesthesiologists (ASA) performance status, stage of colorectal cancer and length of postoperative hospital stay.

### Quality of recovery score (QoR-40)

The QoR-40 is a recovery-specific and patient-rated questionnaire that contains 40 items measuring five dimensions: the physical comfort (12 items), emotional state (nine items), physical independence (five items), psychological support (seven items) and pain (seven items) [8]. The QoR-40 as an assessment of patient-reported outcome, including quality of life, was originally developed and validated in Australia in 2000 [8]. The total score and subscales of the QoR-40 are measured using a five-point Likert scale (for positive items: 1 = none of the time, 5 = all of the time; for negative items, the scoring was reversed) and individual scores are then added together, with the minimum score being 40 points and the maximum score being 200 points. The QoR-40 was specifically designed to measure a patient’s health status after surgery and anesthesia, and its completion time generally ranges from three to 10 min. The Japanese version of the QoR-40 was also validated according to standard methods of cultural adaptation and psychometric analysis in 2011 [12].

### Data collection

Before surgery, usually one or two days before surgery, the QoR-40 Japanese version was explained to the patients. After written informed consent was obtained, QoR-40 questionnaires were completed by patients themselves to determine the preoperative baseline health status. Assistance in completing the QoR-40 was provided if deemed necessary. The QoR-40 were also repeated and collected on postoperative days (POD) 1, 3 and 6 (where the day of surgery = day 0) in the hospital, as well as one month after surgery at an outpatient clinic.

### Statistical analysis

The demographic and perioperative data are presented as medians (25^th^, 75^th^ percentiles), box and whisker plots, or numbers (%), as appropriate. We used the preoperative scores as the baseline health status for the QoR-40, and compared these with the QoR-40 on POD1, 3, 6, and at 1 month. Since the original data of QoR-40 scores were not normally distributed and clearly had a negative skewness, the scores at each point were compared using the Dunn test for nonparametric multiple comparisons. Various factors affected the QoR-40 scores on POD1 compared to the baseline, and these were examined using Wilcoxon’s signed-ranks test. All statistical analyses were performed using the JMP11 software program (SAS institute Japan LTD., Tokyo, Japan). A value of *P* < 0.05 was considered to be significant.

## Results

Ninety-four patients were enrolled in the study. Ninety-three patients completed all QoR-40 questionnaires (five total questionnaires). One patient was unable to complete the postoperative QoR-40 one month after surgery because he had already been discharged and did not come to our outpatient clinic one month after surgery. No patients were withdrawn from the study.

The patient characteristics are shown in Table 1. Overall, 10 patients had one or more postoperative complications (Table 1); there were no deaths. The duration of the postoperative hospital stay was 7.8 ± 4.2 days.

**Table 1:** The patient and perioperative characteristics

		Number	Percent
Gender	Male	61	(65)
	Female	33	(35)
Age	(years)	67.7 ± 11.4	
ASA physical status	I	40	(43)
	II	46	(49)
	III	8	(8)
Stage	I	18	(19)
	II	36	(38)
	III	24	(26)
	IV	16	(17)
operation	open	51	(54)
	laparoscopic	43	(46)
Length of operation (min)		217 ± 72	
Blood loss (gram)		439 ± 511	
Hospital stay (days)		7.8 ± 4.2	
Postoperative complications			
leakage		2	
anastomotic bleeding		1	
postoperative ileus		4	
intraabdominal abscess		1	
pneumonia		1	
cardiac dysfunction		1	

**Fig. 1:**
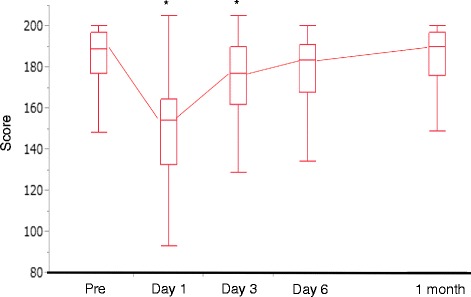
The median (25^th^, 75^th^ percentiles) of perioperative global QoR-40 scores are presented as box and whisker plots. POD; postoperative day. The scores on POD1, POD3, POD6, and at 1 month, were compared to the baseline using Dunn’s test for non-parametric multiple comparisons. * *p* < 0.05

Figure 1 shows the QoR-40 scores as box and whisker plots. The median scores (189, 154, 177, 183.5 and 190), at pre-operation (as baseline), POD1, POD3, POD6 and 1 month after surgery are indicated with horizontal bars. The vertical bars indicate the range and the horizontal boundaries of the boxes represent the first and third quartiles.

The median (25^th^, 75^th^ percentiles) preoperative global QoR-40 scores, indicating the baseline health status, was 189 (176.75, 197) (Fig. 1). On POD1, the global QoR-40 score dropped significantly to 154 (132.5, 164.25) (Fig. 1). On POD3, the score increased to 177 (161.75, 190), but was still significantly lower compared to the baseline (*p* < 0.05, respectively).

However, on POD 6, the global QoR-40 recovered to 183.5 (167.9, 191), which was not significantly different from the baseline level (*p* = 0.06). Moreover, the score at 1 month after surgery were 190 (176, 197), which was almost the same as the baseline score (*p* = 1.00). These results of time-dependent changes clearly demonstrated the rapid recovery of colorectal cancer patients under ERAS, who were almost fully recovered to the baseline levels by POD6, and who continued to improve for at least one month.

The perioperative scores of the five dimensions of the QoR-40 are shown in Table 2. The scores on POD1, POD3, POD6 and at one month were compared to the baseline using Dunn’s test for nonparametric multiple comparisons. In all five dimensions, the scores on POD1 were significantly decreased compared to the baseline (*p* < 0.05) (Table 2). Two dimensions, the emotion state and psychological support, were restored by POD3. In two other dimensions, physical comfort and pain, both scores were significantly worse compared to the baseline even on POD6. However, at one month after surgery, the scores recovered to the baseline in all five dimensions.

**Table 2:** The dimensions of the QoR-40 before and after surgery

	Pre	POD1	POD3	POD6	1 month
Physical comfort (60)	57 (55, 60)	44* (40,50)	51.5* (47, 55)	54* (50, 57)	58 (53, 60)
Emotion state (45)	41.5 (36.8, 44)	35* (28.5, 39.3)	38 (35.6, 41.3)	41 (37, 44)	42 (37.5, 45)
Physical independence (25)	25 (22.8, 25)	11* (8, 15)	20* (15.8, 23)	24 (20, 25)	25 (23, 25)
Psychological support (35)	35 (31, 35)	29* (24, 33.3)	33 (28, 35)	34 (31.8, 35)	34 (29, 35)
Pain (35)	34 (32, 35)	29.5* (26, 32)	31.5* (29, 33)	33* (29, 34)	33 (31, 34)

The maximum score for each dimension is reported in parentheses

The values are the medians (25^th^, 75^th^ percentiles)

*p < 0.05 (compared to the baseline)

**Fig. 2:**
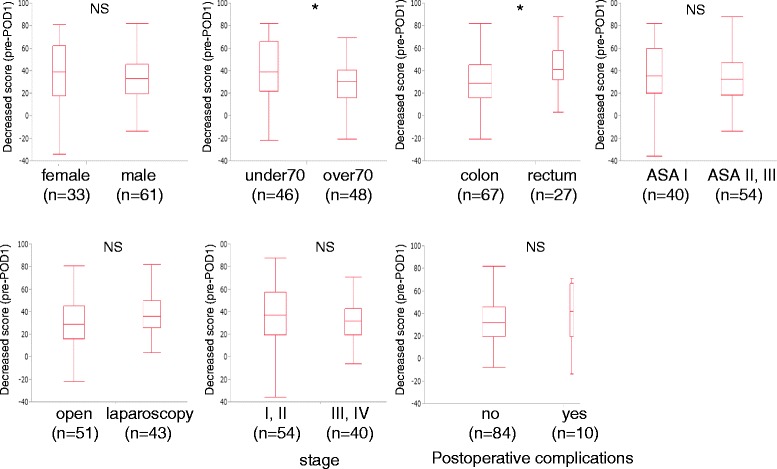
The difference in the QoR scores between POD1 and the baseline, taking into account various factors. The median (25^th^, 75^th^ percentiles) differences between POD1 and the pre-operative values are presented as box and whisker plots.* *p* < 0.05

We also examined the factors that can influence the quality of recovery on POD1. Several factors, such as gender, age (younger than 70 versus older than 70 years), the tumor location (colon versus rectum), the ASA physical status classification (I versus II, III), the surgical approach (laparoscopic versus open), stage of cancer (stage I, II versus III, IV), and occurrence of postoperative complications, were examined to compare the decrease in the QoR40 scores on POD1 from the baseline. As shown in Fig. 2, both the age and tumor location significantly affected the drop on POD1 (*p* < 0.05), with younger patients and rectal cancer patients having worse scores than older patients and colon cancer patients. Interestingly, in addition to gender (*p* = 0.663), the ASA status (*p* = 0.644), stage (*p* = 0.488), surgical approach (*p* = 0.232) and occurrence of postoperative complications (*p* = 0.401) showed no significant influence on the QoR-40 scores on POD1.

## Discussion

The clinical advantages and safety of ERAS protocols have been reported in many studies, but most of these were doctor-reported outcomes. This is the first study of the postoperative patient-reported quality of recovery in colorectal cancer managed under an ERAS protocol using the QoR-40. Our study demonstrated that the QoR-40 scores, which are patient-reported outcomes, of colorectal cancer patients under ERAS management decreased significantly on POD1 but recovered almost to the baseline level on POD6. These results revealed that, in terms of the patient-reported outcomes, colorectal cancer patients under ERAS protocols recover around POD6. In addition, at one month after surgery, all scores of the five dimensions of QoR-40 recovered to the baseline including two dimensions, physical comfort and pain, both of which were significantly worse compared to the baseline even on POD6.

So far, Khan AS et al. [15] have reported the postoperative health-related quality of life of patients managed under an ERAS protocol, using the SF-12 (Short Form 12), which is a questionnaire that evaluates the patient-reported outcome. However, in their study, the SF-12 questionnaires were examined preoperatively, and at two and six weeks after discharge, which may not adequately assess the effectiveness of ERAS during the initial period after surgery. In this study, using the QoR-40 scores, we revealed the effectiveness of ERAS during the initial period after surgery including the time of patient discharge, which we suggest is much more important.

With regard to the quality of recovery after other types of operations which have been assessed using the QoR-40, the outcomes were different from those of colorectal surgery. For example, the quality of health after cardiac surgery was still not restored to the baseline level up to one month after surgery [9]. On the other hand, the global QoR-40 scores after joint replacement (hip or knee arthroplasty) did not show any significant changes compared to baseline even on POD1 [16]. After joint replacement surgery, only the physical independence dimension of the QoR-40 was above the minimal detectable change [16].

Our study also demonstrated that both the patient age and tumor location are significantly associated with the decrease in QoR-40 scores on POD1 compared to the baseline in colorectal cancer patients. That is, younger patients, compared to older patients, and rectal cancer patients, compared to colon cancer patients, had lower QoR-40 scores on POD1. With regard to gender, it has previously been shown that the quality of recovery of patients undergoing general anesthesia for elective surgery differs by with the overall quality of recovery in females being poorer than that in males [17]. In that previous study, the mean patients’ age was 39.5 years old, and premenopausal female sex hormones, such as progesterone, were suggested to affect the quality of recovery [17]. In our study, the patients’ ages were quite different (with females being postmenopausal) so that the gender was not a factor influencing the quality of recovery.

Our study also demonstrated that open surgery and laparoscopic surgery, under ERAS, did not differ in terms of the drop in the QoR-40 scores at POD1 from the baseline. Generally, laparoscopic surgery is considered to be less invasive, and thus is expected to result in better QoR-40 scores than open surgery; however, this was not the case. This result is compatible with a previous study comparing open surgery and laparoscopic surgery with ERAS, which found that patients had a similar health-related quality of life at two and six weeks after surgery [15]. A multicenter randomized controlled trial of conventional versus laparoscopic surgery for colorectal cancer with an ERAS program in the United Kingdom, also showed that patients treated by experienced surgeons according to ERAS had similar physical fatigue and patient-reported outcomes in both groups [18]. Taken these results together, ERAS benefits colorectal cancer patients regardless of surgical approach.

Japan has a unique health insurance system, where there is public health insurance for the whole nation. Thus, patients in Japan are usually not urged to be discharged early after surgery. In Japan, the median LOS is around 10 to 19 days [19]. At Tokyo Metropolitan Bokutoh Hospital, the LOS had been 12 days before the implementation of the ERAS protocols (data not shown). As shown in Table 1, the LOH decreased to seven days after the implementation of ERAS protocols. This QoR-40 study clearly demonstrated that the quality of recovery, as indicated by patient-reported outcomes, agrees with discharge around POD6 for colorectal cancer patients treated under an ERAS protocol.

## Conclusions

This study clearly demonstrated that the postoperative patient-reported quality of recovery using QoR-40 is in agreement with discharge around POD6 for colorectal cancer patients under ERAS.

## Abbreviations

ERAS: Enhanced recovery after surgery
QoR-40: 40-item quality of recovery score
POD: Post-operative day
LOS: Length of hospital stay
SF-36: Short form 36 health survey
QOL: Quality of life
IRB: Institutional review boards
ASA: American Society of Anesthesiologists
